# Determinants of bird species richness, endemism, and island network roles in Wallacea and the West Indies: is geography sufficient or does current and historical climate matter?

**DOI:** 10.1002/ece3.1276

**Published:** 2014-10-02

**Authors:** Bo Dalsgaard, Daniel W Carstensen, Jon Fjeldså, Pietro K Maruyama, Carsten Rahbek, Brody Sandel, Jesper Sonne, Jens-Christian Svenning, Zhiheng Wang, William J Sutherland

**Affiliations:** 1 Center for Macroecology, Evolution and Climate, Natural History Museum of Denmark, University of CopenhagenUniversitetsparken 15, DK-2100, Copenhagen Ø, Denmark; 2 Conservation Science Group, Department of Zoology, University of CambridgeDowning Street, Cambridge, CB2 3EJ, UK; 3 Department of Biological Sciences, Aarhus UniversityNy Munkegade 114, DK-8000, Aarhus C, Denmark; 4 Plant Phenology and Seed Dispersal Group, Departamento de Botânica, Instituto de Biociências, Universidade Estadual Paulista (UNESP)Avenida 24-A n° 1515, Rio Claro, SP, 13506-900, Brazil; 5 Programa de Pós-Graduacão em Ecologia, Universidade Estadual de Campinas (UNICAMP)Cx. Postal 6109, Campinas, SP, 13083-865, Brazil

**Keywords:** Birds, Caribbean, current climate, endemism, historical climate, island biogeography, modularity, species richness, Wallacea, West Indies

## Abstract

Island biogeography has greatly contributed to our understanding of the processes determining species' distributions. Previous research has focused on the effects of island geography (i.e., island area, elevation, and isolation) and current climate as drivers of island species richness and endemism. Here, we evaluate the potential additional effects of historical climate on breeding land bird richness and endemism in Wallacea and the West Indies. Furthermore, on the basis of species distributions, we identify island biogeographical network roles and examine their association with geography, current and historical climate, and bird richness/endemism. We found that island geography, especially island area but also isolation and elevation, largely explained the variation in island species richness and endemism. Current and historical climate only added marginally to our understanding of the distribution of species on islands, and this was idiosyncratic to each archipelago. In the West Indies, endemic richness was slightly reduced on islands with historically unstable climates; weak support for the opposite was found in Wallacea. In both archipelagos, large islands with many endemics and situated far from other large islands had high importance for the linkage within modules, indicating that these islands potentially act as speciation pumps and source islands for surrounding smaller islands within the module and, thus, define the biogeographical modules. Large islands situated far from the mainland and/or with a high number of nonendemics acted as links between modules. Additionally, in Wallacea, but not in the West Indies, climatically unstable islands tended to interlink biogeographical modules. The weak and idiosyncratic effect of historical climate on island richness, endemism, and network roles indicates that historical climate had little effects on extinction-immigration dynamics. This is in contrast to the strong effect of historical climate observed on the mainland, possibly because surrounding oceans buffer against strong climate oscillations and because geography is a strong determinant of island richness, endemism and network roles.

## Introduction

Island biogeography has long fascinated biologists and contributed much to our understanding of the processes that determine species diversity (Darlington 1957; MacArthur and Wilson 1967; Terborgh 1973; Lack 1976; Case et al. 1983; Ricklefs and Lovette 1999; Triantis et al. 2008; Whittaker et al. 2008). In their theory of island biogeography, MacArthur and Wilson (1967) recognized that island species richness was determined by extinction-immigration dynamics, Wilson (1961), Ricklefs and Cox (1972), and Ricklefs and Bermingham (2002) further explained the role of taxon cycles in determining speciation and horizontal and vertical changes in species distributions over time. Despite this, island biogeography has given little attention to historical factors, such as historical climate, which could influence the distribution of endemic species (Emerson and Kolm 2005; Whittaker et al. 2008; Steinbauer et al. 2012; Weigelt et al. 2013; Cabral et al. 2014). Instead, island biogeography has mainly focused on correlations between species richness and island geography, that is, current area, elevation, and isolation, as well as current climate (MacArthur and Wilson 1967; Wright 1983; Kalmar and Currie 2006; Kreft et al. 2008; Whittaker et al. 2008).

These studies, often focused on birds, have shown that geography is a strong determinant of species distribution on islands. Specifically, large islands are known to have more species, both nonendemics and endemics, than small islands (Gotelli and Abele 1982; Whittaker et al. 2008; Tjørve 2009), and island elevation is known to be associated with habitat heterogeneity and therefore increased number of species, especially of endemics that often are found on interior mountain tops (Ricklefs and Cox 1972; Ricklefs and Lovette 1999; Ricklefs and Bermingham 2002; Jønsson et al. 2014). An island's distance to the nearest mainland may also affect its richness as not all bird families are able to disperse to the islands furthest away from the mainland (Terborgh 1973), causing remote islands to have fewer species, especially fewer nonendemics (Kalmar and Currie 2006); however, in some cases endemics may reach higher numbers far away from the mainland as isolated islands provide better opportunities for speciation (Whittaker et al. 2008). Recently, it was shown that isolation measures integrating mainland and intra-island distances perform better in explaining island diversity than distance to the mainland, indicating that intra-island colonization and source-sink dynamics are important for species distributions on islands (Weigelt and Kreft 2013). Also contemporary climate may affect bird distributions. Notably high temperature and precipitation may provide productive environments enabling more species to coexist (Wright 1983; Hawkins et al. 2003).

Continental and global analyses show that although total bird species richness may be explained well by topography and current climate (Hawkins et al. 2003), contemporary factors are less successful at explaining patterns of endemism, that is, richness of species with small to moderate geographic range-size (Fjeldså et al. 1999; Dynesius and Jansson 2000; Jansson 2003; Jetz et al. 2004; Rahbek et al. 2007; Sandel et al. 2011). Small-ranged endemic species often have poor dispersal abilities and fail to occupy otherwise suitable areas, which make them particularly vulnerable when climates change (Svenning and Skov 2004; Sandel et al. 2011). Communities that historically have experienced large changes in climate may therefore have experienced high levels of extinction and low levels of re-colonization, especially of small-ranged endemics. This was recently supported by Sandel et al. (2011), who confirmed the hypothesis that global patterns of amphibian, bird, and mammal endemism are low in areas that experienced considerable climate-change during the late Quaternary (Fjeldså et al. 1999; Dynesius and Jansson 2000; Jansson 2003). Therefore, several competing factors – that is, geography, current climate, and historical climate – may affect species distribution on islands, but the role of historical climate remains largely untested (Cabral et al. 2014).

In addition to island-wide patterns of species richness and endemism, islands may differ in their role in connecting the fauna across an archipelago, which may also be influenced by island geography and climate (Carstensen et al. 2012, 2013). For instance, the geographic position (i.e., isolation) of an island affects its ability to interconnect the fauna of other islands within the archipelago. Species distributions across an island system can be treated as an island-species network, and the detection of modules within this biogeographical network based on species distributions has been proposed as a promising approach to identify tightly linked islands and species, that is, faunal or floristic subregions within an archipelago (Carstensen and Olesen 2009; Carstensen et al. 2012; Thébault 2013; Kougioumoutzis et al. 2014). The network approach also scores islands accordingly to their topological position in the island-species network, that is, their biogeographical role, which may reflect how individual islands contribute to biogeographical connectivity and source-sink dynamics, both locally within modules and across an archipelago (Carstensen et al. 2012). It has been shown that island area, elevation, and isolation are related to island's biogeographical role, but how current and historical climate affect biogeographical roles remains untested (Carstensen et al. 2012). Similarly, it has been hypothesized, but not tested, that species richness and endemism affect island's biogeographical role (Carstensen et al. 2012).

Here, we use native terrestrial breeding birds in Wallacea and the West Indies to: (1) evaluate whether geography adequately determines the distribution of species on islands or, alternatively, current climate and Quaternary climate-change has left detectable imprints on bird species distributions within equatorial island systems. Specifically, we test the hypothesis that endemic species are found particularly on islands having experienced the least historical climate-change; and (2) assess whether island geography, current climate, historical climate-change or species distribution patterns best explain an island's role in determining biogeographical modules, that is, avian subregions within each archipelago. Specifically, we aim at evaluating the hypothesis that islands responsible for defining biogeographical modules are rich in endemic species, whereas those islands acting as stepping stones between faunal subregions/modules are rich in nonendemics, as proposed by Carstensen et al. (2012). By comparing two archipelagos, we are able to evaluate whether the identified biogeographical patterns and processes are affected by regional history (Ricklefs 2004) or repeat themselves in independent island systems.

## Materials and Methods

### Species richness and endemism

We gathered species distribution data for native terrestrial breeding birds on islands in Wallacea and the West Indies. We excluded marine and freshwater dependent species (e.g., ducks, pelicans, shorebirds, and herons) and nonbreeding migratory species whose distribution patterns are likely to be determined by factors other than island features (Kalmar and Currie 2006). We focused on contemporary species distributions; however, if known, we included recently extinct species (last four hundred years) and excluded species introduced by humans. For Wallacea, we followed the taxonomy of the International Ornithologists' Union World Bird List (http://www.worldbirdnames.org/), and for the West Indies, we followed the taxonomy of the American Ornithologists' Union checklist (http://checklist.aou.org/). Compared to the datasets used in Carstensen et al. (2012, 2013), we conducted a major update of the bird distribution of Wallacea, and for both archipelagos, we updated the taxonomy (as of February 2014).

**Figure 1:**
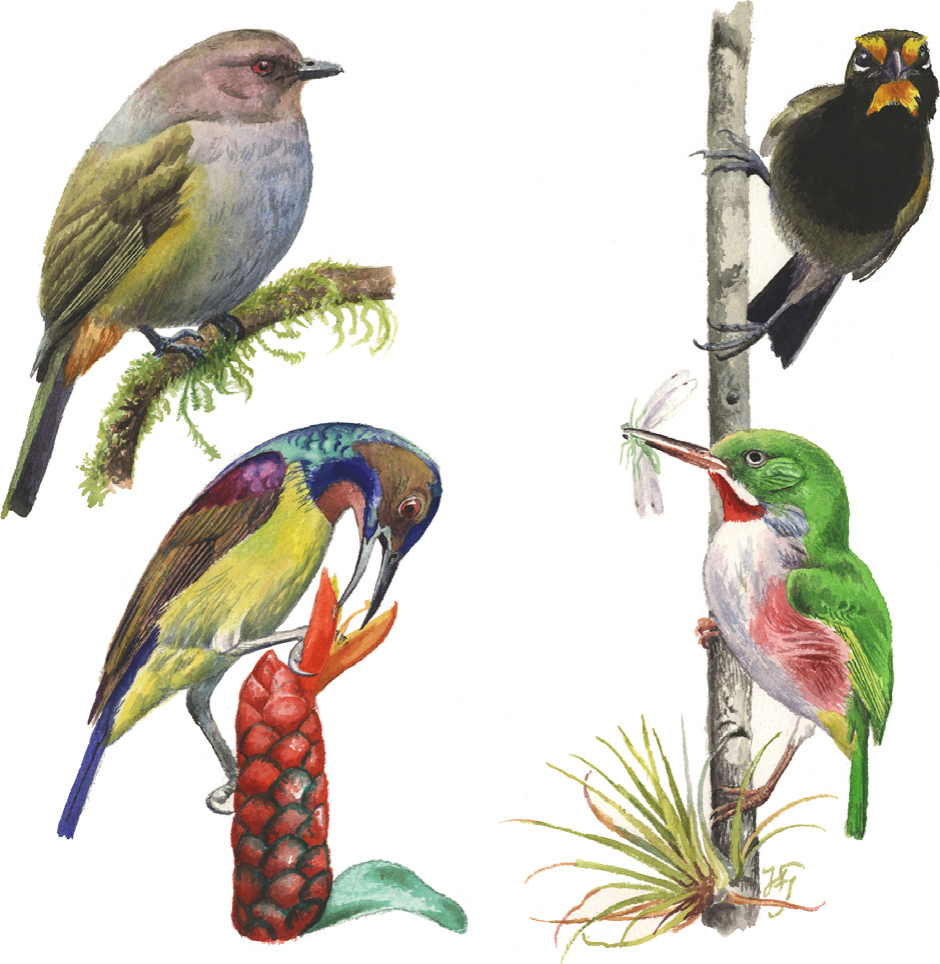
Four bird species representing a nonendemic and an endemic species to Wallacea (left) and the West Indies (right). Upper left: Olive-flanked Whistler (*Hylocitrea bonensis*) endemic to Sulawesi, the largest island in Wallacea. Lower left: Brown-throated Sunbird (*Anthreptes malacensis*), a nonendemic species found on Sulawesi and other islands in the western part of Wallacea and throughout much of South-East Asia. Upper right: Yellow-faced Grassquit (*Tiaris olivacea*) is a nonendemic West Indian species mainly found on large Greater Antillean islands, or nearby satellite islands, and in Central America and the northwestern South America. Lower right: Narrow-billed Tody (*Todus angustirostris*) is endemic to Hispaniola, the second largest and the most mountainous islands of the West Indies. Illustrations by Jon Fjeldså.

**Figure 2:**
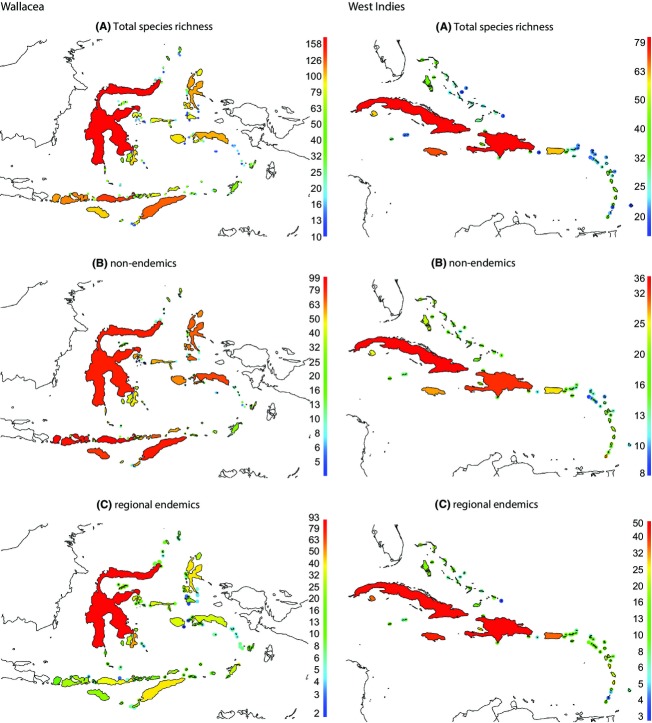
Map of Wallacea (left) and the West Indies (right) showing (A) total species richness, (B) richness of nonendemics, and (C) richness of regional endemics. Notice the logarithmic scale.

Inspired by continental studies examining historical effects on species distributions (e.g., Rahbek et al. 2007; Sandel et al. 2011), we not only used total species richness but also divided species into subgroups according to their degree of endemism. In continental grid-based analyses, this is achieved by dividing species into range-size quartiles (e.g., Rahbek et al. 2007) or using a range-size threshold (Sandel et al. 2011), whereas we here adopted a division that is more suited for the island biogeographical context. For each archipelago, we calculated per-island: (1) total species richness; (2) richness of nonendemic species, that is, species whose breeding range extends beyond Wallacea and the West Indies (Fig.1). The vast majority of these nonendemic species have colonized the islands from surrounding source mainland and have not originated within the regions; (3) richness of regional endemics, that is, species only breeding in Wallacea/West Indies (Fig.1). These are species that probably originated within the archipelagos, but their distributions within each archipelago may also be determined by colonization and extinction dynamics (Whittaker et al. 2008). Our aim was to make a subdivision that is as comparable as possible between the two archipelagos and that reflects a gradient from the *ecological* time-scale (nonendemics) to presumably increasing focus on *historical* or *evolutionary* time-scales (regional endemics). In total, our datasets comprised 119 islands and 513 native breeding land birds in Wallacea and 57 islands and 243 native breeding land birds in the West Indies (Appendix S1; Fig.2). In both archipelagos, we Log_10_-transformed the total species richness and richness of nonendemics and regional endemics.

### Island biogeographical roles

Based on species distributions, for each archipelago, we constructed an island-bird presence/absence matrix and used a network approach to detect biogeographical modules and island roles (as in Carstensen et al. 2012). Modules were defined as subunits of highly connected nodes within the network and were detected using an optimization algorithm that maximizes modularity (Marquitti et al. 2014). We used simulated annealing as the optimization algorithm, but in contrast to previous studies using this approach in biogeographical network studies (e.g., Carstensen and Olesen 2009; Carstensen et al. 2012, 2013; Kougioumoutzis et al. 2014), we calculated a modularity metric appropriate for a bipartite matrix such as an island-species matrix consisting of both islands and species (Thébault 2013). The biogeographical modules and the modularity metric were computed using the software MODULAR, and the observed level of modularity was contrasted with two benchmark null models to assess its significance (Marquitti et al. 2014). After defining the biogeographical modules, we calculated two scores that are related to an island's biogeographical role within an archipelago (Carstensen et al. 2012). The first score, the local topological linkage, *l*, (often named the standardized within-module degree) reflects how well an island is connected within its own module relative to other islands in its module. It is calculated as:


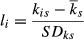


where *k*_*is*_ is the number of links of node *i* to other nodes in its own module *s*; 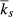 and *SD*_*ks*_ are the average and standard deviation of the within-module *k* of all nodes in *s*. The second score, the regional topological linkage, *r*, (often named the among-module connectivity) reflects how an island within a module is connected with respect to other modules, and is defined as:


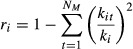


where *k*_*i*_ is the number of links of *i*; and *k*_*it*_ is the number of links from *i* to nodes in module *t* (including *i*'s own module). When calculating the local topological linkage for each island, only the number of links to the other islands was considered, excluding links of birds to islands (for a similar approach, see Carstensen et al. 2013).

### Environmental predictors

#### Geographical predictors

As potential predictors of total species richness and endemism categories, we used the “classic” island geographic features: island area (km^2^), maximum elevation (m), and distance to nearest source mainland (km). For Wallacea, we used Australia, New Guinea, and Borneo as potential extra-Wallacean colonization “mainland” sources (Carstensen and Olesen 2009; Michaux 2010; Jønsson et al. 2012), whereas for the West Indies, we used North America, Central, and South America as mainland sources. Isolation effects may also depend on the distance to large source islands within an archipelago. Hence, in addition, we estimated the distance from a target island to nearest mainland or large island, using the island size threshold of 10,000 km^2^ previously shown to be important (Weigelt and Kreft 2013). For all measures of distance, we used Google Earth to determine the distance (km) between the coastline of the target island to the coastline of nearest mainland or large island. If the isolation estimates that integrate mainland and intra-island distances perform better than the distance to mainland, it indicates that intra-island colonization is important for current patterns of species distributions. Area and elevation were taken from the literature; these were supplemented with estimates based on the 500 m digital elevation model from the Consortium for Spatial Information (CGIAR [http://srtm.csi.cgiar.org/], derived from the Shuttle Radar Topography Mission data; Farr et al. 2007) to determine the island area (sum of the area of all land pixels constituting the island) and maximum elevation for those islands that we could not find information for in the literature. In both archipelagos, island area and elevation were Log_10_-transformed to improve the assumptions of normality/linearity and to make our study comparable with previous island biogeography studies that did not consider climate predictors. Isolation measures were left untransformed (for range and mean values, see Appendix S1).

#### Current and historical climate predictors

For extraction of current climate predictors, we used WorldClim 30-arc second climate products (Hijmans et al. 2005) to calculate the mean annual temperature (MAT) and precipitation (MAP) for each island. For the historical climate, we estimated both Quaternary climate-change anomaly and velocity of MAT and MAP since the Last Glacial Maximum (LGM) as putative historical climate predictors (Appendix S1). Estimates of MAT and MAP at the LGM were obtained from the CCSM3 model and were statistically downscaled to a 2.5-arc minute resolution (Collins et al. 2006; Otto-Bliesner et al. 2006). We determined the average annual rate of change in MAT or MAP for each 30-arc second grid cell on each island since the LGM, yielding a temporal climate gradient, and calculated the slope of the current MAT or MAP surface at all cells as obtained from the WorldClim 30-arc second climate products (Hijmans et al. 2005), giving a measure of the spatial gradient. Our MAT and MAP Quaternary climate anomaly estimates were equal to the temporal climate gradient described above (i.e., LGM climate minus current climate, absolute values), whereas dividing the temporal gradient by the spatial gradient gives the climate-change velocity since the LGM. Hence, Quaternary climate-change velocity incorporates both spatial and temporal gradients of climate to estimate the local displacement rate of climate conditions (Loarie et al. 2009; Sandel et al. 2011). We summarized an island's overall MAT/MAP anomaly and MAT/MAP velocity by calculating the mean within each island (Appendix S1). For all analysis, MAP, MAT anomaly, and MAT/MAP velocities were Log_10_-transformed, whereas MAT and MAP anomaly were untransformed. Due to the geographic extent of the datasets, we did not attempt to estimate other historical factors that may also potentially affect contemporary bird distributions, such as plate tectonics, volcanic history, island age, or Pleistocene sea-level changes (Murphy et al. 2004; Dalsgaard et al. 2007; Kreft et al. 2008; Presley and Willig 2008; Ricklefs and Bermingham 2008; Whittaker et al. 2008; Michaux 2010; Lohman et al. 2011; Jønsson et al. 2012). Hence, our analyses take the pre-Quaternary diversity and distribution as a given and asks how these have responded to subsequent climate change.

### Multiple regression analysis

For each archipelago, and separately for total species richness and richness of nonendemics and endemics, we used ordinary least squares regression and a model selection analysis based on information theory, as outlined by Diniz-Filho et al. (2008), to test the effect of island geography, current climate and historical climate. To avoid problems with multicollinearity, and as temperature and precipitation velocities were strongly correlated with each other, and with elevation and temperature/precipitation anomalies, we excluded temperature and precipitation velocities from all analyses (Appendix S2). We thereafter fitted models with all combinations of the remaining explanatory variables, that is, island area, elevation, isolation to nearest mainland, isolation to nearest large landmass, current precipitation, current temperature, and temperature and precipitation anomalies. For each archipelago, and separately for total species richness and richness of nonendemics and endemics, we used AIC_c_ to identify minimum adequate models (MAMs) (Table1). We report standardized regression coefficients using ordinary least squares regression for both an averaged model based on weighted *w*_*i*_ and for MAMs (Diniz-Filho et al. 2008). We use a similar approach for an island's local, *l*, and regional, *r*, topological linkage level. The variance inflation factor VIF < 2.4 and the condition number CN < 3.2, indicating that multicollinearity was not a problem in any of the MAMs. Finally, we used Pearson correlation to test the association between *l*, *r* and total species richness, richness of nonendemics and endemics. All analyses were conducted using the software Spatial Analysis in Macroecology (SAM) 4.0 (Rangel et al. 2010).

**Table 1:** Models containing island geography, current and historical climate as predictors of total species richness, richness of nonendemics, and richness of regional endemics. The standardized regression coefficients are reported for ordinary least squares regression and reported for both an averaged model based on weighted *w*_i_ and minimum adequate models (Diniz-Filho et al. 2008). We also report AIC_c_ and coefficients of determination (*R*^2^) from partial regression models separating the effect of geography, current and historical climate (Rangel et al. 2010). 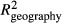 describes total variation explained by island area, elevation and isolation, whereas 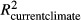 and 
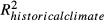
 reflects the additional (i.e., unique) variation explained by current climate and historical climate-change, respectively. The analysis was conducted separately for Wallacea (*n* = 119) and the West Indies (*n* = 57)

	Total species richness			Nonendemic richness			Regional endemic richness		
	Σ *w*_*i*_	Averaged	MAM	Σ *w*_*i*_	Averaged	MAM	Σ *w*_*i*_	Averaged	MAM
Wallacea									
Area	1.00	+0.84	+0.86**	1.00	+0.75	+0.82**	1.00	+0.89	+0.91**
Elevation	0.34	+0.07	–	0.54	+0.13	–	0.29	−0.05	–
Isolation mainland	0.25	−0.01	–	0.28	−0.03	–	0.29	−0.04	–
Isolation landmass	0.89	−0.13	−0.14**	0.98	−0.19	−0.20**	0.31	+0.05	–
MAT	0.26	−0.01	–	0.30	−0.05	–	0.78	+0.18	+0.18*
MAP	0.49	−0.08	–	0.68	−0.15	−0.17**	0.29	−0.05	–
Anomaly MAT	0.26	+0.00	–	0.85	−0.14	−0.17**	0.99	+0.22	+0.21**
Anomaly MAP	0.30	−0.02	–	0.60	−0.13	–	1.00	+0.23	+0.22**
AIC_c_			−79.8			−38.6			−54.3
*R*^2^			0.74			0.72			0.69
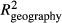			0.74			0.69			0.58
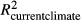			0.00			0.03			0.02
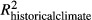			0.00			<0.00			0.07
West Indies									
Area	1.00	+0.67	+0.69**	1.00	+0.64	+0.61**	1.00	+0.55	+0.54**
Elevation	0.83	+0.22	+0.17*	0.22	+0.02	–	0.88	+0.28	+0.22*
Isolation mainland	0.31	+0.01	–	0.26	−0.05	–	0.28	+0.07	–
Isolation landmass	1.00	−0.26	−0.26**	1.00	−0.39	−0.40**	0.32	−0.10	–
MAT	0.40	−0.11	–	0.41	+0.12	–	0.86	−0.27	−0.27**
MAP	0.84	+0.21	+0.19**	0.93	+0.26	+0.26**	0.34	+0.14	–
Anomaly MAT	0.82	−0.16	−0.14*	0.26	−0.05	–	0.84	−0.21	−0.21*
Anomaly MAP	0.30	−0.07	–	0.30	+0.08	–	0.39	−0.14	–
AIC_c_			−136.0			−150.2			−60.8
*R*^2^			0.85			0.81			0.74
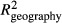			0.82			0.75			0.69
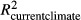			0.02			0.06			0.04
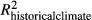			<0.01			0.00			0.03

** *P* < 0.01,

* *P* < 0.05, ^NS^nonsignificant.

## Results

Of the 513 breeding land bird species in our Wallacea dataset, 300 (58%) are endemics of the region and 213 (42%) are nonendemics that also breed outside of Wallacea. Of the 243 breeding species in West Indies, 176 (72%) are endemics and 67 (28%) nonendemics. Hence, Wallacea has considerably higher bird species richness, but proportionally smaller amount of endemics than the West Indies (Fig.2).

In terms of island properties, it is worth noting that the islands in Wallacea on average are considerably smaller but higher, wetter, less isolated, and with higher precipitation anomaly than islands in the West Indies. Otherwise, the mean of island characteristics are broadly similar in the two regions (Appendix S1; for correlations between predictors, see Appendix S2).

### Species richness and endemism

Overall, the explanatory power of our models was higher in the West Indies (*R*^2^ = 74–85%) than in Wallacea (*R*^2^ = 69–74%; Table1). In both archipelagos, the models explained total species richness (*R*^2^ = 74–85%) better than nonendemic richness (72–81%) and endemic richness (69–74%). In the West Indies, high temperature anomaly diminished total species richness and richness of endemics, whereas in Wallacea high temperature anomaly diminished the richness of nonendemics while high temperature and precipitation anomalies increased the richness of endemics. In both archipelagos, the effects of climate anomaly were marginal (0–7%), current climate effects stronger (0–47%) but island geography almost explained as much as the total variation explained (58–82%). In both Wallacea and the West Indies, island area was the single most important variable, increasing the richness of both nonendemics and regional endemics (Table1; Fig.2). The second most important variable was distance to nearest large landmass; in both archipelagos the total richness and richness of nonendemics decreased with increasing distance to a large landmass, whereas endemism was unrelated to isolation. In the West Indies only, richness of endemics was positively related to island elevation. High precipitation increased richness of nonendemics in the West Indies, whereas the opposite was the case in Wallacea (Table1).

### Island biogeographical roles

**Figure 3:**
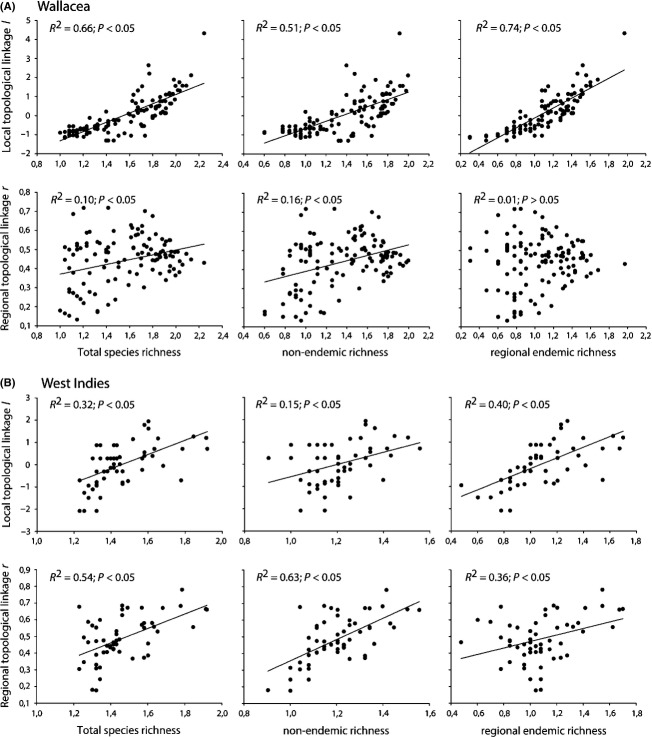
The relationship between island biogeographical network roles (*l* and *r*) and total species richness, richness of nonendemics, and richness of regional endemics for (A) Wallacea and (B) the West Indies. Notice that in both archipelagos local topological linkage *l* correlated strongest with richness of regional endemics, whereas regional topological linkage *r* correlated strongest with richness of nonendemics.

As previously reported by Carstensen et al. (2012) both archipelagos were modular; Wallacea had four modules (*Q*_*B*_ = 0.434; p_null1_ < 0.001; p_null2_ < 0.001), and the West Indies had six modules (*Q*_*B*_ = 0.360; p_null1_ < 0.001; p_null2_ < 0.001; See Appendix S3 for details). In both archipelagos, island geography (11–71%) was overall a more important determinant of local and regional topological linkage than current (0–32%) and historical climate (0–25%). Specifically, islands with high local topological linkage, *l*, were large islands situated far from large landmasses (Table2). The regional topological linkage, *r*, was mainly associated with isolation. In both archipelagos, distance to the nearest mainland increased the regional topological linkage level, whereas the effect of distance to nearest landmass differed between the two regions (Table2). Elevation was essentially unrelated to both the local and regional topological linkage level. High current temperature decreased local and increased regional topological linkage in the West Indies, whereas current climate was unimportant in Wallacea. High precipitation anomaly increased the regional topological linkage level in Wallacea; historical climate was unrelated to topological linkage level in the West Indies. In both archipelagos, endemic richness was strongly correlated with an island's local topological linkage (40–74%), whereas nonendemic richness correlated strongly with an island's regional topological linkage (16–63%; Fig.3).

**Table 2:** Models containing island geography, current and historical climate as predictors of island biogeographical network roles. The standardized regression coefficients are reported for ordinary least squares regression and reported for both an averaged model based on weighted *w*_i_ and minimum adequate models (Diniz-Filho et al. 2008). We also report AIC_c_ and coefficients of determination (*R*^2^) from partial regression models separating the effect of geography, current and historical climate (Rangel et al. 2010). 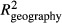 describes total variation explained by island area, elevation and isolation, whereas 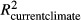 and 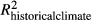 reflects the additional (i.e., unique) variation explained by current climate and historical climate-change, respectively. The analysis was conducted separately for Wallacea (*n* = 119) and the West Indies (*n* = 57)

	Wallacea						West Indies					
	Within module degree *l*			Among module connectivity *r*			Within module degree *l*			Among module connectivity *r*		
	Σ *w*_*i*_	Averaged	MAM	Σ *w*_*i*_	Averaged	MAM	Σ *w*_*i*_	Averaged	MAM	Σ *w*_*i*_	Averaged	MAM
Area	1.00	+0.86	+0.91**	0.64	+0.19	+0.13^NS^	0.45	+0.23	+0.26^NS^	0.73	+0.27	+0.30*
Elevation	0.48	−0.12	−0.12^NS^	0.30	+0.03	–	0.39	+0.20	–	0.24	−0.04	–
Isolation mainland	1.00	−0.22	−0.22**	0.66	+0.15	+0.13^NS^	0.29	−0.13	–	0.92	+0.34	+0.33**
Isolation landmass	0.67	+0.11	+0.10^NS^	0.78	+0.18	+0.20*	0.59	+0.22	+0.26*	1.00	−0.76	−0.76**
MAT	0.33	+0.06	–	0.40	+0.13	–	0.98	−0.52	−0.44**	0.67	+0.26	+0.28*
MAP	0.48	+0.09	–	0.33	+0.10	–	0.28	−0.12	–	0.99	+0.58	+0.60**
Anomaly MAT	0.37	+0.06	–	0.29	+0.04	–	0.36	−0.15	–	0.24	+0.01	–
Anomaly MAP	0.46	+0.08	+0.10^NS^	1.00	−0.50	−0.47**	0.32	−0.14	–	0.25	−0.04	–
AIC_c_			199.2			−180.3			138.4			−114.3
*R*^2^			0.72			0.32			0.39			0.67
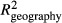			0.71			0.11			0.29			0.48
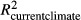			0.00			0.00			0.10			0.09
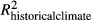			<0.01			0.21			0.00			0.00

** *P* < 0.01,

* *P* < 0.05, ^NS^nonsignificant.

## Discussion

### Determinants of species richness and endemism

We show that Quaternary climate-change and contemporary climate have relatively little effect on bird distributions within Wallacea and the West Indies. Moreover, these effects were idiosyncratic to each archipelago. In the West Indies, endemics occurred marginally less frequently on islands with high Quaternary climate-change, whereas the opposite was the case in Wallacea (Table1). This indicates that within tropical island systems, such as Wallacea and the West Indies, Quaternary climate-change has had little effect on bird distributions. This is in contrast to the continental setting where high Quaternary climate-change is strongly associated with low endemism (Sandel et al. 2011), suggesting contrasting effects of Quaternary climate-change on the distributions of small-ranged island and mainland endemic birds.

Why are endemic land birds not consistently underrepresented on islands that have experienced high Quaternary climate-change? Quaternary climate-change has been suggested to cause incomplete range-filling and species extinctions of small-ranged species, thereby linking global patterns of Quaternary climate-change and endemism (Svenning and Skov 2004; Sandel et al. 2011). Based on our results, we suggest that the same may not apply to endemics on islands, at least not within tropical islands, such as Wallacea and the West Indies. First of all, according to taxon cycle theory, island species go through stages of expansion and contraction that may be more closely related to co-evolutionary relations with predators, parasites, and competitors than to glacial climate cycles (Ricklefs and Bermingham 2002). Further, the oceanic islands of Wallacea and the West Indies are surrounded by thermally very stable sea currents and are therefore climatically relatively stable (Steele 1985, Cronk 1997; Kissling et al. 2012; Dalsgaard et al. 2013), at least compared to those parts of the world having low levels of endemism (Sandel et al. 2011; Weigelt et al. 2013), and Quaternary climate-change may simply have been too minor to cause significant extinctions of endemic birds in Wallacea and the West Indies. Finally, island species may respond to low species richness and interspecific competition by showing density compensation, becoming highly abundant with generalized niches (Diamond 1975; Ricklefs and Cox 1978; Olesen et al. 2002; Dalsgaard et al. 2009), and may therefore be less vulnerable to changing climates than species on the mainland. Taken together, the discrete nature of island systems, the tropical setting surrounded by water buffering against climate-change, density compensation, generalized niches, and taxon cycles could explain why contemporary distributions of endemics in Wallacea and the West Indies appear to be largely unrelated to historical climate-change.

Alternatively, Quaternary climate-change may have caused endemics in Wallacea and the West Indies to occupy less suitable climate niches – depressing their abundances – and contracted their geographic range within islands, but without causing a significant number of local extinctions. Hence, there may be an undetected extinction debt of endemics, as has also been proposed for high mountain endemic plants in the Alps (Dullinger et al. 2012). In addition, in view of the complex interaction between sea currents, coastlines, and topography in these archipelagos, the modeled Quaternary climate-change may not fully describe the actual climate stability pattern. Only future climate models better incorporating these effects, especially the effect of sea currents, may reveal whether model uncertainly in predicting historical climate has affected our results. In addition, hurricanes cause a large amount of environmental disturbance in the West Indies (Presley and Willig 2008) and much extinction on islands can be related to human activity, including introduction of non-native species, hunting, and current land use (Pregill and Olson 1981; Ficetola and Padoa-Schioppa 2009; Duncan et al. 2013), which may obscure a potential imprint of the modeled Quaternary climate-change. Finally, although contemporary climate seems more important than the modeled Quaternary climate-change in determining species distributions in Wallacea and the West Indies, this does not, *per se*, prove that geographic patterns of species occurrence are determined by contemporary climate. For instance, it is noteworthy for Wallacea that there are multiple kinds of empirical and modeled evidence to suggest that the same major warm sea current has, since the mid-Miocene, passed south through the Makassar strait (west of Sulawesi), then east to the Banda sea and south around Timor, causing constantly high humidity in flanking mountains, while other parts of the region had variable and more seasonal climates (Hall et al. 2011). This could explain the long persistence of several phylogenetically old endemics, especially in the mountains of western Sulawesi. However, irrespectively of possible extinction debt, climate model uncertainty, and correlations between past and current climate, it is clear that island geography explained more and more consistently the distribution of land birds in Wallacea and the West Indies.

The influence of island area, elevation and isolation on species richness, and endemism patterns in Wallacea and the West Indies largely corroborate previous island biogeography studies. In both archipelagos, the observed strong positive effect of island area on total species richness and richness of both nonendemics and endemics – as well as the importance of topography for endemics in the West Indies – may be attributable to an array of mechanisms, including greater resources/energy, habitat and climate diversity, topographic heterogeneity, higher potential for speciation, and lower extinction rates on large and mountainous islands (MacArthur and Wilson 1967; Fjeldså et al. 1999; Ding et al. 2006; Rahbek et al. 2007; Kreft et al. 2008; Whittaker et al. 2008). This may to some extent also reflect the fact that communities on large and mountainous islands can track changing climates vertically (Fjeldså et al. 1999; Fjeldså and Irestedt 2009; Sandel et al. 2011) and are better protected against environmental disturbance, for instance in the West Indies by providing refugia during hurricanes (Presley and Willig 2008). Area and elevation may therefore be proxies for many factors, including climate. The apparent insignificant role of topography in Wallacea may be caused by island area and elevation being positively correlated (Appendix S2), and hence, we suggest that both island area and topography are important, as observed in the West Indies.

As in a global island biogeographical study on plant species richness (Weigelt and Kreft 2013), the distance to the nearest large landmass, not just the distance to the nearest source mainland outside of each archipelago, affected bird species distribution in both archipelagos. In both Wallacea and the West Indies, large distances to the nearest large landmass diminished total species richness and nonendemics, illustrating that distance is a limiting factor for nonendemic species dispersing across an archipelago. This also suggests that nonendemics use large islands as stepping stones to reach smaller islands. Surprisingly, there were not more endemics far from the nearest source mainland, that is, we did not find evidence that diversification rates are highest on distant islands where few lineages colonize (Whittaker et al. 2008). In Wallacea this may be attributable to islands in the center, currently and for millions of years, being drier and climatically more unstable than those at the western and southern fringe (Hall et al. 2011; Appendix S2). Additionally, the region has a highly complex geological history, and linked complex species dispersal histories and intra-archipelago species-pumps (Jønsson et al. 2012, 2014), which may potentially obscuring any simple measure of isolation. For instance, the southern Moluccas are geologically linked with New Guineas Vogelkop peninsula (Hall 2002), and the large and geologically highly complex island of Sulawesi in the north-western fringe of Wallacea has high *in situ* speciation (Lohman et al. 2011) and could therefore have served as an intra-archipelago species-pump for the surrounding islands.

### Determinants of island biogeographical network roles

In both Wallacea and the West Indies, island biogeographical network roles related strongly to species richness/endemism and to island geography. As for species richness and endemism patterns, current and historical climate played only a relatively minor role idiosyncratic to each archipelago. In both archipelagos, local topological linkage, *l*, correlated strongly with richness of endemics, whereas regional topological linkage, *r*, associated best with richness of nonendemics, as hypothesized by Carstensen et al. (2012). Moreover, high *l* islands were large islands situated far from other large islands, indicating that high *l* islands potentially act as speciation pumps and source islands for surrounding smaller islands, defining biogeographical modules, that is, avian subregions consisting of many local “module” endemics (Carstensen et al. 2012). On the other hand, high *r* islands were in both archipelagos situated distantly from the mainland and were in Wallacea characterized by unstable climates; they may be sink islands acting as stepping stones for widespread and good dispersing nonendemic species whose breeding range extends across biogeographical module borders (Carstensen et al. 2012).

## Conclusions

We conclude that island area, elevation, and isolation are the most important factors explaining bird distributions on islands, having strong effects on patterns of species richness, endemism and island network roles. Quaternary climate-change, on the other hand, only influences island network roles and the distribution of island endemics to a small degree, at least within archipelagos surrounded by thermally stable tropical seas. However, we cannot rule out that island endemics due to poor ability to cross water currently occupy unsuitable climate niches, that is, island endemics may have experienced declining populations and there may even be an extinction debt of island endemics. Still, taken together with some other results also showing a weak effect of historical climate change on island communities (Kissling et al. 2012; Dalsgaard et al. 2013), it does indicate that oceans may partly buffer island communities from global warming (Cronk 1997), although less vagile organism such as amphibians and plants may be more susceptible to climate change (Sandel et al. 2011; Weigelt et al. 2013; Cabral et al. 2014). Future global scale island biogeographical studies or studies of archipelagos from other parts of the world, notably, from high latitude archipelagos which have experienced considerable higher Quaternary climate-change than Wallacea and the West Indies (Weigelt et al. 2013) are needed to confirm the generality of our findings.

## Supporting Information

**Appendix S1.** Island properties (range and mean) in the datasets of Wallacea and the West Indies. **Appendix S2.** Correlations between predictor variables in Wallacea and West Indies. **Appendix S3.** Additional figure S1 illustrating the biogeographical modules in Wallacea and the West Indies.
