# Supplementary material for: Determinants of bird species richness, endemism, and island network roles in Wallacea and the West Indies: is geography sufficient or does current and historical climate matter?
Source: Ecol Evol. 2014 Oct 2;4(20):4019–31. doi: 10.1002/ece3.1276 (PMC4242583; doi:10.1002/ece3.1276)
Supplement: Supplementary file 1 [file ece30004-4019-SD1.doc]

**Appendix S1.** Island properties (range and mean) in the datasets of Wallacea and the West Indies.

| **Predictor variables** | **Wallacea** | **West Indies** |
| --- | --- | --- |
| No. islands | 119 | 57 |
| Area (km^2^) | 6-174,167 (2930) | 13-105,007 (3968) |
| Maximum elevation (m) | 22-3726 (716) | 8-3098 (494) |
| Distance, nearest mainland (km) | 114-860 (492) | 103-1022 (604) |
| Distance, nearest landmass > 10,000 km^2^ (km) | 1-361 (81) | 1-692 (314) |
| MAT (°C) | 23.2-27.3 (26.1) | 23.8-27.0 (25.6) |
| MAP (mm) | 1046-3601 (1994) | 602-2599 (1296) |
| Anomaly MAT, absolute (°C) | 1.7-2.7 (2.1) | 1.3-4.1 (1.9) |
| Anomaly MAP, absolute (mm) | 15-1370 (466) | 67-700 (345) |
| Velocity MAT (m/yr) | 0.07-10.95 (1.58) | 0.02-11.25 (3.23) |
| Velocity MAP (m/yr) | 0.05-23.87 (3.36) | 0.11-18.63 (3.26) |

**Appendix S2.** Pearson’s correlations between predictor variables in Wallacea (above diagonal) and the West Indies (below diagonal).

|  | Area | Elevation | Isolation  mainland | Isolation  landmass | MAT | MAP | Anomaly MAT | Anomaly MAP | Velocity MAT | Velocity MAP |
| --- | --- | --- | --- | --- | --- | --- | --- | --- | --- | --- |
|  | (km^2^) | (m) | (km) | (km) | (°C) | (mm) | (°C) | (mm) | (m/yr) | (m/yr) |
| Area (km^2^) | – | +0.72^*^ | +0.07^NS^ | +0.08^NS^ | -0.65^*^ | -0.05^NS^ | +0.05^NS^ | -0.18^NS^ | -0.32^*^ | -0.26^*^ |
| Elevation (m) | +0.25^NS^ | – | +0.19^†^ | -0.12^NS^ | -0.78^*^ | +0.05^NS^ | -0.07^NS^ | +0.02^NS^ | -0.65^*^ | -0.38^*^ |
| Isolation mainland (km) | -0.38^†^ | +0.02^NS^ | – | -0.17^NS^ | -0.28^†^ | -0.20^†^ | +0.12^NS^ | +0.04^NS^ | -0.14^NS^ | -0.17^NS^ |
| Isolation landmass (km) | -0.35^†^ | +0.27^†^ | +0.19^NS^ | – | +0.28^†^ | +0.15^NS^ | -0.28^†^ | -0.05^NS^ | -0.00^NS^ | -0.17^NS^ |
| MAT (°C) | -0.68^*^ | -0.36^*^ | +0.36^†^ | +0.20^NS^ | – | +0.08^NS^ | +0.13^NS^ | +0.11^NS^ | +0.56^*^ | +0.53^*^ |
| MAP (mm) | +0.24^†^ | +0.61^*^ | -0.52^*^ | +0.13^NS^ | -0.47^*^ | – | -0.39^†^ | +0.67^†^ | -0.04^NS^ | +0.43^†^ |
| Anomaly MAT (°C) | +0.15^NS^ | -0.36^†^ | -0.50^†^ | -0.28^†^ | -0.16^NS^ | -0.08^NS^ | – | -0.16^NS^ | +0.22^*^ | -0.16^NS^ |
| Anomaly MAP (mm) | +0.15^NS^ | +0.65^*^ | +0.12^NS^ | -0.14^NS^ | -0.26^NS^ | +0.52^*^ | -0.15^NS^ | – | +0.05^NS^ | +0.67^*^ |
| Velocity MAT (m/yr) | +0.30^†^ | -0.74^*^ | -0.35^†^ | -0.47^†^ | +0.05^NS^ | -0.37^†^ | +0.56^†^ | -0.47^†^ | – | +0.44^*^ |
| Velocity MAP (m/yr) | +0.17^NS^ | -0.72^*^ | -0.05^NS^ | -0.48^†^ | +0.17^NS^ | -0.55^†^ | +0.47^†^ | -0.28^†^ | +0.84^*^ | – |

^*^ P < 0.05 both when using non-spatial statistics and when significance level is based on degrees of freedom corrected for spatial auto-correlation using Dutilleul’s (1993) method; ^†^ P < 0.05 when using non-spatial statistics, but non-significant when using Dutilleul’s (1993) method; ^NS^ non-significant.

**Appendix S3**. Figure S1. Biogeographical modules in (a) Wallacea and (b) the West Indies. Each colour corresponds to a biogeographical module. In Wallacea we detected four modules as in Carstensen *et al.* (2012), whereas we in the West Indies detected six modules. Compared to Carstensen *et al.* (2012) the West Indies was therefore split up into further two modules: (1) Puerto Rico and Mona islands (blue module) were split from Jamaica and Hispaniola and its satellite islands (yellow module), and (2) the Lesser Antilles was split into two modules, one module consisting of the northern and outer arc younger low-lying islands (red) and a southern inner arc module consisting of Guadeloupe and all main volcanic islands south to Grenada (green module). These splits fit well with island geography and the geological history of the West Indies (Ricklefs & Bermingham, 2008).
